# Pioneering Use of Ionic Liquid‐Based Aqueous Biphasic Systems as Membrane‐Free Batteries

**DOI:** 10.1002/advs.201800576

**Published:** 2018-08-08

**Authors:** Paula Navalpotro, Catarina M. S. S. Neves, Jesus Palma, Mara G. Freire, João A. P. Coutinho, Rebeca Marcilla

**Affiliations:** ^1^ Electrochemical Processes Unit IMDEA Energy Institute Avda. Ramón de la Sagra 3 28935 Móstoles Spain; ^2^ CICECO ‐ Aveiro Institute of Materials Chemistry Department University of Aveiro 3810‐193 Aveiro Portugal

**Keywords:** aqueous biphasic systems, aqueous immiscible electrolytes, membrane‐free batteries, organic redox molecules

## Abstract

Aqueous biphasic systems (ABS) formed by water, ionic liquids (ILs), and salts, in which the two phases are water rich, are demonstrated here to act as potential membrane‐free batteries. This concept is feasible due to the selective enrichment of redox organic molecules in each aqueous phase of ABS, which spontaneously form two liquid‐phases above given concentrations of salt and IL. Therefore, the required separation of electrolytes in the battery is not driven by an expensive membrane that hampers mass transfer, but instead, by the intrinsic immiscibility of the two liquid phases. Moreover, the crosscontamination typically occurring through the ineffective membranes is determined by the partition coefficients of the active molecules between the two phases. The phase diagrams of a series of IL‐based ABS are characterized, the partition coefficients of several redox organic molecules are determined, and the electrochemistry of these redox‐active immiscible phases is evaluated, allowing appraisal of the battery performance. Several redox ABS that may be used in total aqueous membrane‐free batteries with theoretical battery voltages as high as 1.6 V are identified. The viability of a membrane‐free battery composed of an IL‐based ABS containing methyl viologen and 2,2,6,6‐tetramethyl‐1‐piperidinyloxy as active species is demonstrated.

## 1 Introduction

Aqueous biphasic systems (ABS) are ternary systems formed by water and two water‐soluble compounds, such as two polymers, a polymer and a salt, two salts, an ionic liquid (IL) and a salt, or an IL and a polymer. These ternary systems form two liquid phases above given concentrations of the phase‐forming components, in which the two phases are water rich. Due to their water‐rich environment, they have been widely studied as separation platforms of labile molecules.^1^, ^2^ In addition to more conventional polymer‐based systems, which are restricted in terms of their phases' polarities,^3^ IL‐based ABS received significant attention in the last decade since they have shown to display a higher performance in terms of extraction efficiency and selectivity.^4^, ^5^ They have been investigated in the separation of proteins and enzymes,^6^, ^7^, ^8^, ^9^ metals,^10^, ^11^, ^12^ and synthetic drugs,^13^, ^14^ namely by their selective partition between the two liquid phases. In the IL‐based ABS field, the effect of various salting‐out agents, the ILs nature, as well as the operating conditions, such as pH and temperature, upon the phase diagrams behavior and partition of target compounds has been evaluated.^4^, ^7^ There are different strategies to manipulate the molecules/products partition and their selectivity between the coexisting phases of an IL‐based ABS: i) by changing the nature of the phase‐forming components (e.g., polymers, salts, or ILs) and/or by using additives; ii) by changing the composition of the phase‐forming components and phases' composition; and iii) by manipulation of other conditions, such as pH and temperature.^5^

Recently, our group has developed an innovative concept of membrane‐free battery based on the immiscibility of two redox electrolytes; one of them being aqueous and the other one nonaqueous.^15^ Differently from conventional batteries (Li‐ion, Na‐ion, NiMH, etc.) in which the active materials are solids attached to metallic current collectors, in redox flow batteries (RFB) the active species, generally based on metals, are dissolved into the liquid electrolytes which are separated by an ion selective membrane to avoid their mixture.^16^ In our pioneering concept of membrane‐free battery, the use of a separator is not necessary because the electrolytes are immiscible and are phase‐separated by their intrinsic thermodynamic nature.^15^ Moreover, scarce and expensive metallic active compounds, such as vanadium salts, were substituted by organic molecules such as quinones, which are abundant species, less expensive, and environmental friendly. It is important to remark that our strategy is radically different from other reported membrane‐less batteries approaches. For instance, membrane‐less batteries applied to microfluidic designs rely on hydrodynamic engineering to exploit the laminar flow of electrolytes^17^, ^18^ whereas our membrane‐free concept relies on the spontaneous separation of immiscible phases ruled by thermodynamics. Other examples are related with so‐called single flow or semiliquid RFBs in which one of the active species is a solid material such as Zn, lithium, or graphite. In those hybrid systems it is not possible to fully decouple power and energy, being one of the most important advantage of RFBs.^19^ Girault and co‐workers^20^ reported an ion transfer battery utilizing the Galvani potential difference between liquid–liquid interphases in a nonaqueous/aqueous/nonaqueous triphasic system. Unlikely, our membrane‐free battery is based on two immiscible phases and the voltage of this battery exclusively depends on the difference of redox potential of active species dissolved in catholyte and anolyte, as in any type of RFB.

Besides the immiscibility of the two electrolytes, i.e., an aqueous and a nonaqueous phase, in the previous work^15^ we anticipated that the selective solubility of each target redox molecule in the two liquid phases is of paramount importance to develop batteries with minimized crossover. The crossover or crosscontamination of active species from catholyte to anolyte and vice versa reduces the battery performance decreasing its efficiency and provoking a misbalance of active species in the two battery compartments. This crossover has become a severe drawback in nonaqueous RFB where separators are used instead of ion‐selective membranes that are ineffective in these media. Therefore, any approach to mitigate this effect will result in batteries with higher efficiencies and longer cycle‐life.

Here, we propose the use of IL‐based ABS to develop membrane‐free batteries, but contrarily to our previous work^15^ these systems comprise two aqueous‐rich phases, with appropriate electrochemical performance and minimal crosscontamination controlled by the selective partition of the positive and negative redox compounds between the two coexisting phases. To achieve this goal, we carefully tailored the IL‐based ABS in terms of their phases' compositions and nature of ILs used. The partition of target organic redox molecules between the two phases and electrochemistry of each phase were determined. We anticipate the used of IL‐based ABS containing different pairs of organic molecules as membrane‐free batteries just by connecting the two immiscible phases through an external electrical circuit.

## 2 Results

### 2.1 Phase Diagrams and Organic Redox Molecules Partitioning

**Figure 1:**
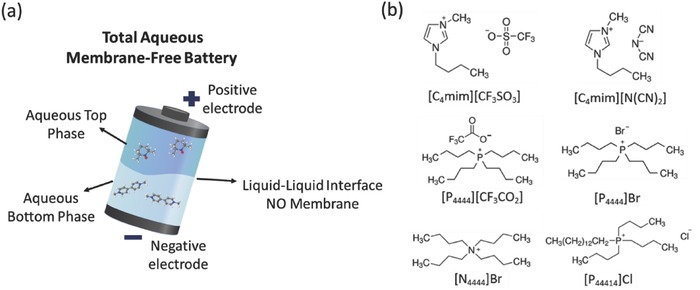
a) Scheme of the membrane‐free battery comprising two aqueous‐rich phases. b) Chemical structure of ionic liquids chosen to form ABS.

In order to study the viability of the application of ABS as the basis for a membrane‐free battery, the main requirement of this battery concept, the immiscibility of the electrolytes, was first investigated. To this end, the possibility of forming two‐phase systems by mixing water, Ils, and Na_2_SO_4_ was addressed. A scheme of the membrane‐free battery comprising two aqueous immiscible phases is depicted in **Figure** **1**a. The phase diagrams of several ABS containing different ILs were determined in this work, whereas others were taken from the literature.^21^ The inorganic salt Na_2_SO_4_ was selected due to its moderate salting‐out ability to induce the formation of IL‐based ABS. Moreover, the acidic‐neutral pH of most of ABS obtained with this salt brings additional advantages, such as the higher chemical and electrochemical stability of some organic molecules, the low corrosive character of the media, and the lower environmental impact in case of electrolyte leak in the battery. Figure 1b depicts the chemical structure of the different ILs investigated to prepare ABS. Since Na_2_SO_4_ is a moderate salting‐out species, ILs with a more hydrophobic character are required to be able to form two‐phase systems in aqueous media.^22^, ^23^, ^24^ Therefore, the ILs chosen are composed of cations with low hydrogen‐bond acidity^25^ and anions of low hydrogen‐bond basicity.^26^ As well‐established in the literature, the toxicity of ionic liquids highly depends on their chemical structure.^27^ Hence, tetraakylammonium and phosphonium‐based ILs have been mainly investigated due to their lower toxicity, low‐cost, and high ability to form ABS.^8^, ^28^, ^29^ In fact, we have previously shown that the ILs affinity for water addressed by their hydrogen‐bond basicity and acidity correlates well with the ABS formation ability.^24^, ^30^ Since it is the salt that induces the salting‐out of the IL leading to the creation of a second liquid phase, the higher the hydrogen‐bond basicity and acidity of a target IL, the lower its capacity to undergo phase separation.

**Figure 2:**
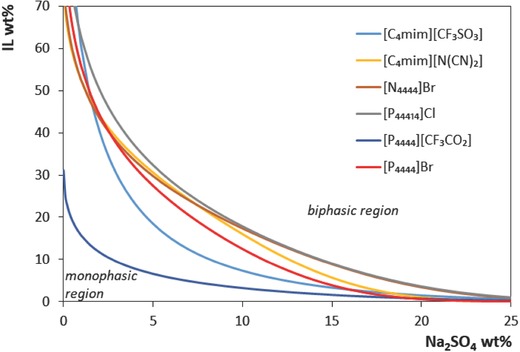
Phase diagrams in weight percentage shown according to the fitting of Equation (1) to the experimental data obtained at 25 °C and atmospheric pressure. Phase diagrams for [C_4_mim][CF_3_SO_3_] and [C_4_mim][N(CN)_2_] were taken from literature.^21^

The binodal curves in weight fraction percentage (kg of IL or salt *per* kg of the other two species, salt or IL + water) for the different ILs are given in **Figure** **2**. Compositions of IL and salt above each binodal curve result in two phase systems, while compositions below result in homogeneous solutions. The water content corresponds to the amount required to reach 100 wt%. The detailed data in weight fraction for the phase diagrams and their correlation by Equation (1) are given in the Supporting Information (Tables S1 and S2, respectively, Supporting Information). From Figure 2, the ability to form ABS, when [IL] = [salt] in weight fraction, follows the order: [P_4444_][CF_3_CO_2_] > [C_4_mim][CF_3_SO_3_] > [P_4444_]Br > [C_4_mim][N(CN)_2_] > [P_44414_]Cl ≈ [N_4444_]Br. This trend is in accordance with the ILs affinity for water, in which both the cation and anion play a synergetic role as explained above. Among the investigated ILs, [P_4444_][CF_3_CO_2_] presents the largest biphasic region, thus requiring lower amounts of salt to be salted‐out from aqueous media. This IL was here designed to prepare a water soluble ionic liquid of high hydrophobicity by combining a cation with low charge density and strong steric shielding with an anion of low hydrogen bond basicity, according to the discussion above. Tie‐line length (TLL) and phases composition for a given composition of initial mixture; IL, Na_2_SO_4_, and H_2_O (35, 10, and 55 wt%, respectively), were calculated accordingly to Equations (2)–(6) and included in Table S3 in the Supporting Information. It should be remarked that the top phase is the IL‐rich phase in all cases, excepting for the ABS containing [C_4_mim][CF_3_SO_3_] in which an inversion of the phases' densities occurs. The pH of each system is also given in Table S3 in the Supporting Information. The systems based on [C_4_mim][CF_3_SO_3_], [N_4444_]Br and [P_44414_]Cl exhibit an lightly acid pH close to 4.5 that favors the stability of the redox molecules and a suitable electrochemical stability window of the electrolytes for the redox activity of the active species, as will be discussed later.

In RFBs, the redox active molecules should be highly soluble in the electrolyte, chemical, and electrochemical stable and should exhibit fast and reversible electrochemical activity. In addition, to achieve a propitious battery behavior, the difference between the redox reaction potentials of the active species should be as large as possible. It must be stressed that the crossover of one redox molecule from one electrolyte to the opposite one (through the membrane in conventional RFB) causes its contamination, and as consequence decreases the battery performance. Accordingly, besides the imperative formation of the two immiscible phases, the transformation of an ABS to a membrane‐free battery requires the highly selective separation of two different redox molecules, one in each phase. As it was mentioned before, the specific solubility of a certain molecule in each phase of an ABS, directly related with the partition coefficients, relies on the physicochemical properties of the phases. Factors such as nature of IL and salt, pH, or temperature affect the partition coefficients in ABS.^4^, ^7^ Here, we analyze the effect of different ILs on the partition coefficients of five organic redox molecules widely investigated in RFB: methyl viologen (MV), hydroquinone (H_2_Q), athraquinone‐2‐sulfonic acid (AQ2S), quinoxaline (QUI), and 2,2,6,6‐tetramethyl‐1‐piperidinyloxy (TEMPO).

**Figure 3:**
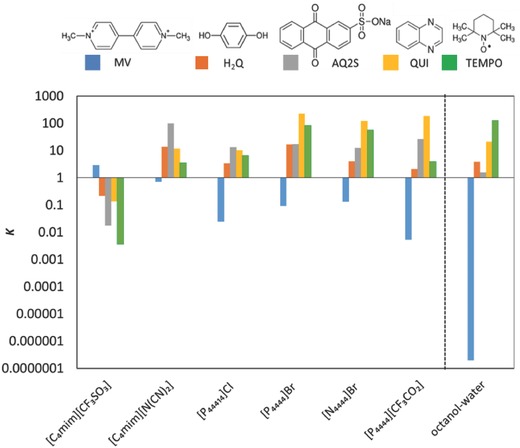
Partition coefficients (*K* = [molecule]_top phase_/[molecule]_bottom phase_) of the target molecules in ABS based on different ionic liquids and the respective octanol‐water (*K*_ow_) partition coefficients taken from ref. 36.

**Figure** **3** represents the partition coefficients (*K*) calculated according to Equation (7) as the ratio of concentrations of each compound in the top phase to that of the bottom phase of ABS composed of Na_2_SO_4_, IL, and water (10, 35, and 55 wt%, respectively). The chemical structures of the redox molecules and their octanol‐water partition coefficients (*K*_ow_)^31^ are also given in Figure 3. Table S4 in the Supporting Information includes the numerical values of partition coefficients (*K*). Values of *K* higher than 1 mean that the target molecule is dissolved preferably in the top phase, whereas values lower than 1 indicate that the target molecule majorly partitions to the bottom phase. As can be seen in Figure 3, a selective separation enabling a top‐phase rich in one redox molecule and a bottom‐phase rich in other molecule was attained in all systems. As mentioned above, the top phase is the IL‐rich phase in all cases, excepting for the ABS containing [C_4_mim][CF_3_SO_3_]. Therefore, it is demonstrated that most of the molecules exhibit higher affinity to the IL‐rich phase whereas MV is selectively partitioned into the salt‐rich phase in all the studied ABS. This means that MV has lower affinity to hydrophobic phase that in this system is the IL‐rich phase. This trend is in close agreement with their octanol‐water partition coefficients, in which only MV has *K*_ow_ values < 1, and thus preferentially partitions to hydrophilic phases, in this work the salt‐rich phase. In the case of systems based on more hydrophobic ILs, such as those containing ILs with phosphonium cations, and particular when combined with the fluorinated anion ([P_4444_][CF_3_CO_2_]), a large increase in the partition coefficients of MV to the salt‐rich phase was observed, further supporting our strategy of designing and synthesizing a more hydrophobic, still water‐soluble, IL.

The separation of the target molecules can be evaluated by selectivity (*S*), defined as the ratio of partition coefficients of two target molecules, which describes their equilibrium distribution between the two immiscible phases. The more different from 1 the selectivity values are, the greater separation between those molecules is achieved. From the partition coefficient determination it was observed that MV is the only molecule that preferentially partitions into the salt‐rich phase. The selectivity values (*S*) of the different molecules with respect to MV are presented in Figure S1 and Table S5 in the Supporting Information. The selectivity values are higher (>100) for all the molecules in those systems in which the ILs are based on the phosphonium cation. For TEMPO and AQ2S especially large *S* values are observed when fluorinated anions are involved, such as in the case of [C_4_mim][CF_3_SO_3_] and [P_4444_][CF_3_CO_2_]. Higher *S* values are obtained with more hydrophobic IL, closely correlating with their ability to form ABS with Na_2_SO_4_—a trend that results from the higher polarity difference between the coexisting phases and the partition being dominated by the hydrophobic interactions as suggested by the dominating role that the *K*_ow_ has on the partition of the molecules. In summary, the ABS comprising the novel and hydrophobic IL [P_4444_][CF_3_SO_3_] is the one that presents the highest selectivity to separate MV from the remaining organic molecules.

### 2.2 Electrochemical Characterization

The theoretical voltage of an IL‐based ABS operating as a membrane‐free battery was determined by the difference between the redox potentials of the two organic molecules selectively dissolved in each phase. Among the tested redox organic molecules, MV was the only one which was selectively separated from the others. This implies that MV should be necessarily one of the two redox molecules incorporated in the IL‐based ABS. The most suitable candidate for the opposite phase should exhibit high partition coefficient in that phase as well as a separated redox potential with respect to MV. Therefore, the electrochemical properties of IL‐ABS containing pairs of redox molecules that selectively migrate to opposite phases were investigated.

**Figure 4:**
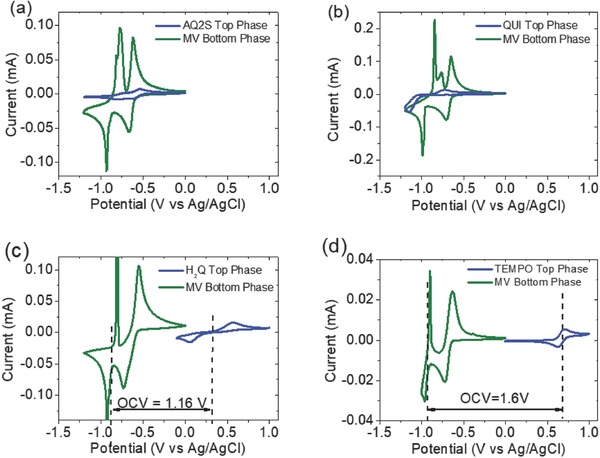
CV of each phase for the system based on P_44414_Cl + Na_2_SO_4_ with different active species at 20 × 10^−3^ m concentration a) MV + AQ2S, b) MV + QUI, c) MV + H_2_Q, and d) MV + TEMPO.

Due to the commercial availability and low price of [P_44414_]Cl in comparison with other ILs, the ABS composed by this IL was selected as case study to investigate the electrochemistry of all possible combination of redox couples. Other physicochemical aspects of this [P_44414_]Cl‐based ABS such as its intermediate pH and its wide electrochemical stability window were also taken into consideration. Four different redox ABS formed by [P_44414_]Cl were prepared by adding together 20 × 10^−3^ m solutions of MV and AQ2S, MV and QUI, MV and H_2_Q, and MV and TEMPO to the original ternary mixture. Then, the electrochemical performance of each phase was separately tested by cyclic voltammetry (CV) in three‐electrode electrochemical cells. Results are given in **Figure** **4**.

**Figure 5:**
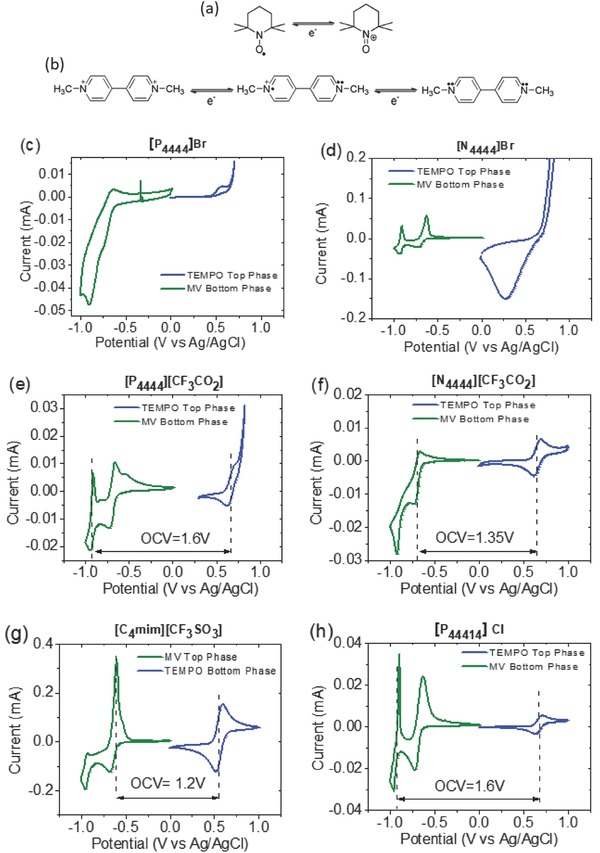
Redox reactions of the active species: a) TEMPO b) methyl viologen. Cyclic voltammetry of the phases separately of the systems based on IL + Na_2_SO_4_ + 20 × 10^−3^ m TEMPO‐20 × 10^−3^ m MV. c) [P_4444_]Br. d) [N_4444_]Br. e) [P_4444_][CF_3_CO_2_]. f) [N_4444_][CF_3_CO_2_]. g) [C_4_mim][CF_3_SO_3_]_._ h) [P_44414_]Cl. Scan rate 10 mV s^−1^. MV (in green) TEMPO (in blue).

The bottom phase being rich in MV in the four systems displays a CV curve with two reversible and intense redox peaks at negative potentials which corresponds to the reversible reduction‐oxidation of MV molecule. This reaction occurs in two steps exchanging two electrons in total. The first step corresponds to the formation of the radical cation and the second one corresponds to the formation of the neutral form (see **Figure** **5**b). Therefore, the bottom phase rich in MV would act as the anolyte or negative electrolyte of the battery. The top phases rich in AQ2S (Figure 4a) and QUI (Figure 4b) also show reduction and oxidation peaks in the CVs but they appear at similar redox potential than for MV, meaning that the resultant battery would have a very low voltage. In addition, and although according to the partition coefficients the presence of AQ2S or QUI in the bottom phase is relatively small, it still causes some distortions in the redox signal of MV‐rich bottom phase. Contrary to AQ2S and QUI, top phases rich in H_2_Q and TEMPO exhibit reversible redox behavior at positive potentials (Figure 4c,d). At negative potentials, when analyzed in the whole voltage range, these top phases do not show significant electrochemical activity due to the small presence of MV. Similarly, the bottom phase does not exhibit relevant redox activity at positive potentials (see Figure S2, Supporting Information). Both systems offer a redox potential sufficiently different from MV for being considered as catholyte or positive electrolyte when combined with MV. In fact, the theoretical voltages of membrane‐free batteries formed by MV/H_2_Q and by MV/TEMPO would be as high as 1.16 and 1.6 V, respectively. Table S6 in the Supporting Information shows a detailed analysis of electrochemical characterization of the different redox ABS.

On account of the higher redox potential, partition coefficients, and selectivity of TEMPO compared with H_2_Q, TEMPO was selected as redox pair for the catholyte to be combined with MV anolyte for the remaining IL‐based ABS. In fact, the combination of these two redox molecules has been recently explored for aqueous redox flow batteries with organic redox compounds.^32^, ^33^ Thus, the electrochemical characterization of different redox ABS containing these two molecules was carried out. Redox‐active ABS containing different ILs were prepared by adding TEMPO and MV (20 × 10^−3^ m concentration) as active species within the mixture. After formation of the biphasic system and succeeding migration of TEMPO and MV to the IL‐rich and salt‐rich phase, respectively, the immiscible redox phases were electrochemically characterized in three‐electrode electrochemical cells (Figure 5). For comparison purposes, the electrochemical behavior of the immiscible phases without active species is gathered in Figure S3 in the Supporting Information. CVs of [P_4444_]Br‐based ABS represented in Figure 5c are very distorted and did not display a reversible electrochemical activity in any of the two phases. At negative potentials the anolyte exhibits an intense reduction peak (−0.85 V vs Ag/AgCl) which is likely due to the stripping process caused by the low solubility of the neutral specie of MV, which is a common issue in the viologen family.^34^ The water electrolysis that might provoke hydrogen evolution at this potential in acidic electrolytes was discarded by CV of the blank electrolyte (see Figure S3a, Supporting Information). Concerning to the catholyte behavior, a large and irreversible oxidation peak appears at 0.8 V versus Ag/AgCl, being likely associated with the oxidation of Br^−^ anion from the ionic liquid to Br_2_, as corroborated by control experiment, Figure S3a in the Supporting Information. Although partition coefficients and selectivity of MV and TEMPO redox molecules are appropriate, this ABS is not a good candidate as membrane‐free battery due to the parasitic reactions occurring in both catholyte (top phase) and anolyte (bottom phase).

Figure 5d shows the CVs of the [N_4444_]Br‐based ABS with two reversible redox peaks at negative potentials (−0.67 and −0.91 V) related with the two step MV redox reaction depicted in Figure 5b. However, the CV of the IL‐rich top phase shows that the oxidation of Br^−^ to Br_2_ still occurs at positive potentials due to the presence of Br^−^ anion in the IL (see CV of control experiment in Figure S3b in the Supporting Information). As a result, this system is not suitable for being used as a membrane‐free battery and an IL having a different counter‐anion should be employed to form redox active ABS.

Thermodynamic and electrochemical aspects of [P_4444_][CF_3_CO_2_]‐based ABS in which Br^−^ of the IL was substituted by [CF_3_CO_2_]^−^, were also investigated. Figure 3 shows that the partition coefficient of MV was enhanced, whereas the partition coefficient of TEMPO decreased, resulting in similar values of selectivity in the two IL‐ABS (Figure S1, Supporting Information). The electrochemical analysis of [P_4444_][CF_3_CO_2_]‐based ABS depicted in Figure 5e shows a quite reversible response associated with the two steps redox reaction of MV in the anolyte, whereas in the catholyte the redox activity of TEMPO is overlapped with an irreversible oxidation process occurring at 0.75 V. This peak was attributed to the oxygen evolution occurring during water electrolysis in electrolytes with basic pH such as in this system (pH 11.6 as indicated in Table S3, Supporting Information). This was corroborated by the CV of the control sample in Figure S3c in the Supporting Information. By contrast, as observed in Figure S3d in the Supporting Information, the oxygen evolution is not favored in acidic [N_4444_][CF_3_CO_2_]‐based ABS (pH 2.7) resulting in good electrochemical stability of top phase TEMPO‐catholyte as evidenced in Figure 5f. The redox reaction of TEMPO corresponds to the oxidation to its oxoammonium cation in which one electron is exchanged, as described in Figure 5a. The CV of the bottom phase shows an intense reduction peak at −0.92 V attributed to the irreversible reduction of the MV to its neutral specie which shows lower solubility.^34^ In order to avoid this irreversibility, the battery voltage should be cutoff after the first step redox reaction of MV. In such case, only one electron per MV molecule, the one exchanged in the first reaction step MV^2+^/MV^+^, would be utilized in the battery.

Electrochemical response of the system based on [C_4_mim][CF_3_SO_3_] with pH 4.6 is shown in Figure 5g. A pair of well‐defined and totally reversible peaks at 0.55 V (vs Ag/AgCl) were obtained for TEMPO top‐phase without any signal of water decomposition. The response of MV in the bottom phase exhibits two pairs of peaks at −0.62 and at −0.93 V. It should be mentioned that, the second step of the reaction shows poor reversibility with an oxidation peak less intense than the reduction peak. This irreversibility might be attributed to the stripping process of the neutral specie of MV, as it was commented before.^34^ Similarly to the previous case, only the first step of the MV reaction would be exploited effectively in a battery. It is worth mentioning that this behavior is intrinsical to the MV molecule and most examples of RFB with this molecule in literature describe the use of a cutoff voltage.^33^

In Figure 5h the CVs of the biphasic system based on [P_44414_]Cl are plotted. A reversible redox peak at 0.67 V was observed corresponding to the reaction of TEMPO in the IL‐rich top phase. In this system, the two steps associated with MV redox reaction in the salt‐rich bottom phase appear at −0.68 and −0.93 V with quite reversible redox behavior. In this example, the two electrons involved in the redox reaction of MV might be utilized in a battery doubling their theoretical capacity.

As mentioned above, the theoretical open circuit voltage (OCV) of a battery can be estimated as the difference between the two redox potential at which each reaction take place in each immiscible phase. Therefore, in all of the evaluated IL‐based ABS the OCVs values are between 1.2 and 1.6 V, the latter being one of the highest OCV reported for an aqueous organic redox flow battery.^32^, ^33^, ^35^, ^36^, ^37^, ^38^, ^39^

According to the thermodynamic and electrochemical properties of the investigated ABS, those containing [N_4444_][CF_3_CO_2_], [C_4_mim][CF_3_SO_3_], and [P_44414_]Cl fulfill all the requirements to be transformed in total aqueous membrane‐free batteries. These requirements include appropriate partition coefficients for MV and TEMPO between the two phases, high selectivity, good electrochemical stability of both top and bottom phases, and reversible electrochemistry of MV and TEMPO active molecules, resulting in a high theoretical open circuit voltage of 1.35, 1.2, and 1.6 V, respectively. Particularly interesting is the case of [P_44414_]Cl with the higher OCV and the higher electrochemical reversibility of the MV‐based anolyte. Therefore, we selected this redox [P_44414_]Cl‐based ABS containing TEMPO and MV to assemble a full battery just by immersing two carbon electrode in each of the two phases and without using any membrane or separator (see Figure S4, Supporting Information). The suitable conductivity of the redox electrolytes is ensured by the concentration of ionic liquid in the top phase (catholyte) and salt in the bottom phase (anolyte) which are around 1.2 and 1.8 m, respectively, as can be obtained from Table S3 in the Supporting Information. Ionic conductivities of top and bottom phases were found to be 8.77 and 101.7 mS cm^−2^, respectively.

**Figure 6:**
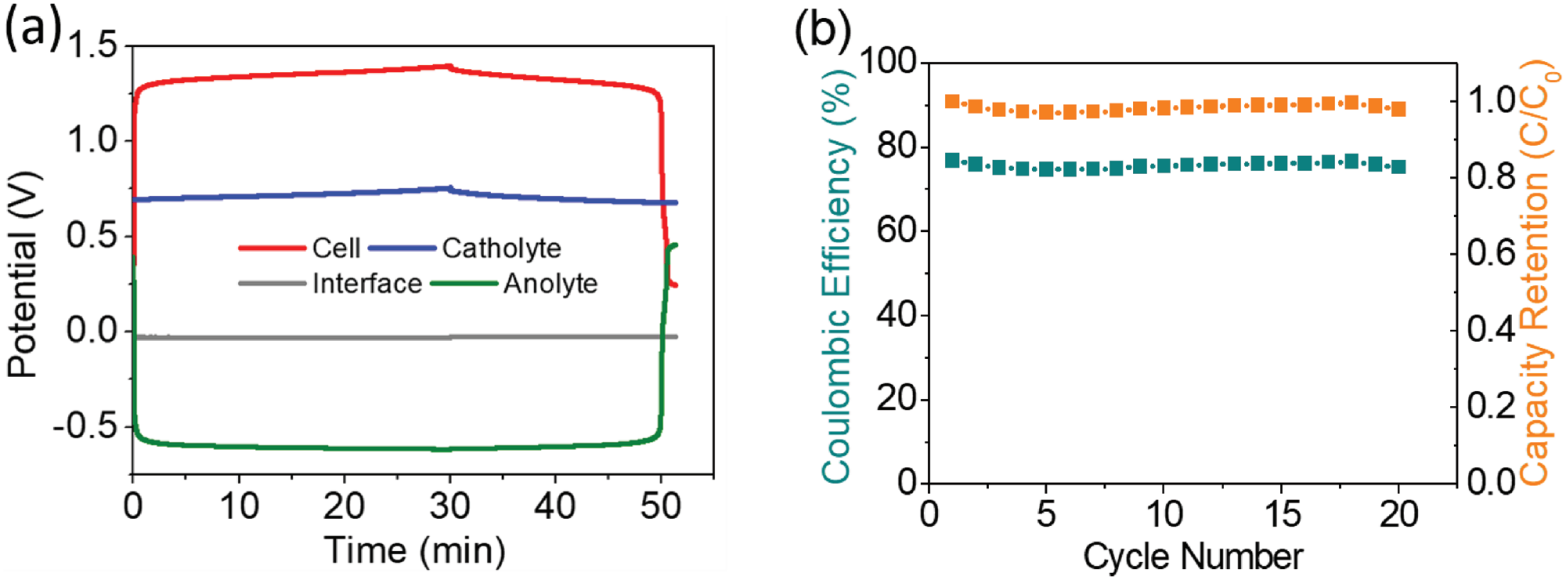
a) Galvanostatic charge–discharge cycle of an aqueous [P_44414_]Cl‐based battery at 0.16 mA cm^−2^. b) Cycling stability over 20 cycles.

Initially, the electrolytes are at discharge state, thus, the battery was charged up to 20 % state of charge (SOC) at 0.16 mA cm^−2^ and then, discharged completely at the same current density. **Figure** **6**a depicts this galvanostatic charge–discharge cycle showing that the battery shows very stable plateaus for both charging and discharging at 1.35 V and exhibiting very low ohmic drop. This voltage is close to the one anticipated by the CV experiment considering that during the battery operation only the first step of the MV reaction occurred, as confirmed by the potential of the anolyte during the cycle. The individual potential profiles of the catholyte (top phase), the anolyte (bottom), and the interface are very flat presenting very low overpotentials with potential values remaining stable at 0.7 V, −0.62 V, and 25 mV, respectively. The area specific resistance was extracted from the ohmic drop of the battery at the end of the discharge. The obtained value of 25 Ω cm^2^ is quite high probably due to the large distance between the electrodes that is expected to be reduced in an optimized cell design. The low overpotential of the interface contrasts with the high overpotential shown by ion‐selective membranes in conventional RFBs which contribute significantly to the overall resistance of the battery. The coulombic efficiency (CE) displayed by the battery is around 70% due to an inherent self‐discharge process of the membrane‐free battery. This phenomenon consists of a direct chemical reaction between the generated specie in each phase that diffuses and meets each other at the interface coming back to their original state. Thus, the crossover of the active species in full reduced/oxidized state does not take place, allowing for keeping the active species concentration in each phase invariant. On the other hand, it should be pointed out that, under these operational conditions, the results show a membrane‐free battery with high voltage efficiency (VE, 99.8%) that together with the coulombic efficiency (70%) results in a membrane‐free battery with energy efficiency (EE) as high as 70%. Figure S5a in the Supporting Information represents the discharge polarization test of the battery at 20% SOC from 0.16 up to 8 mA cm^−2^. This figure also shows the individual potentials of the electrolytes and the interface endowing the analysis of their individual contribution to the total resistance of the battery. In fact, it is observed that the area specific resistance (ASR) of the battery is mainly influenced by the catholyte which exhibits the major polarization probably due to its higher viscosity and lower ionic conductivity. On the other hand, it should be pointed out that the small contribution to the ASR of the interface (around 7%) compares very positively with other aqueous redox flow batteries in which around the 70% of the total resistance come from the membrane. As can be observed in Figure 6b, the membrane‐free battery exhibited a very stable performance with excellent capacity retention over 20 cycles demonstrating the reliability of the system (Figure S5b, Supporting Information). Although further characterization is being carried out in our group to analyze the whole performance of these new type of membrane‐free battery, these promising results confirm the applicability of IL‐based ABS, liquid–liquid systems in which the two phases are water rich, in the development of aqueous membrane‐free batteries.

## 3 Conclusion

The feasibility of ABS, liquid–liquid systems in which both phases are water rich, as immiscible redox electrolytes for an innovative electrochemical energy storage device, namely a membrane‐free battery, was here demonstrated for the first time. The selective partition of two different organic redox molecules between the phases is crucial for the effective performance of the electrochemical device. It was found that an increase in the hydrophobicity of the ILs used enhances the partition of the redox molecules and selectivity (>100). ABS containing the ILs [N_4444_][CF_3_CO_2_], [C_4_mim][CF_3_SO_3_] and [P_44414_]Cl fulfill all the thermodynamic and electrochemical requirements to be transformed in membrane‐free batteries. The gathered results also confirm that ABS are advantageous to control some key factors, such as the crossmigration of the species in the innovative membrane‐free batteries field. Some of the theoretical battery voltages obtained are higher than those previously reported in organic aqueous RFBs, reinforcing the membrane‐free battery as an up‐and‐coming application for the ABS platform. We demonstrated that the [P_44414_]Cl ‐based ABS containing MV and TEMPO operates as an aqueous membrane‐free battery with voltage of 1.35 V and high values of voltage, coulombic and energy efficiencies. Therefore, we here expanded the concept of membrane‐free battery, initially reported for aqueous/nonaqueous systems, to biphasic systems in which the two phases are aqueous, bringing some advantages in terms of cost, environmental issues and battery performance.

## 4 Experimental Section

*Reagents*: The following ILs were investigated: 1‐butyl‐3‐methylimidazolium dicyanamide ([C_4_mim][N(CN)_2_]) 98%, 1‐butyl‐3‐methylimidazolium triflate ([C_4_mim][CF_3_SO_3_]) 99%, and tributyltetradecylphosphonium chloride ([P_44414_]Cl) > 95% supplied by Iolitec; tetrabutylammonium bromide ([N_4444_]Br) 98% obtained from Fluka; tetrabutylphosphonium bromide ([P_4444_]Br) Cyphos IL163 supplied by Cytec Industries Inc.; and tetrabutylphosphonium trifluoroacetate ([P_4444_][CF_3_CO_2_]) synthesized by us. The reagents used in the synthesis of the last IL were a tetrabutylphosphonium hydroxide ([P_4444_]OH) solution (40 wt% in water), purchased from Sigma‐Aldrich, and trifluoroacetic acid (TFA acid) 99% supplied by Acros Organics. The synthesis procedure comprises an acid–base reaction carried out between [P_4444_]OH and TFA at 60 °C for 2 h. Then, to remove the solvent excess, the sample was subjected to evaporation on a rotary evaporator under vacuum. Finally, the obtained IL was dried at 50 °C under vacuum (0.1 Pa) during 72 h. The purity of the IL was then checked by ^1^H, ^13^C, ^31^P, and ^19^F NMR spectra and found to be >99 wt%. The inorganic salt sodium sulfate anhydrous (Na_2_SO_4_) ACS reagent ≥99% was purchased from Sigma‐Aldrich. The organic molecules TEMPO 98%, anthraquinone 2‐sulfonic acid sodium salt monohydrate 97% (AQ2S), QUI 99%, MV dichloride hydrate 98%, and hydroquinone (H_2_Q) reagent plus >99% were supplied by Sigma‐Aldrich and used as received.

*ABS Phase Diagrams*: In this work, novel ternary systems composed of Na_2_SO_4_ + H_2_O and the following ionic liquids: [N_4444_]Br, [P_4444_]Br, [P_4444_][CF_3_CO_2_], and [P_44414_]Cl, were determined at 25 °C (± 1 °C). The remaining phase diagrams were taken from the literature.^21^, ^40^, ^41^ All phase diagrams were determined at 25 °C and atmospheric pressure using the cloud point titration method.^42^, ^43^ A salt aqueous solution (≈25 wt% for Na_2_SO_4_) and aqueous solutions of the different hydrophilic ILs (ranging from 60–80 wt%, depending on the IL) were prepared. Then, repetitive dropwise addition of the salt solution was added to the IL solution until a cloud solution was detected (biphasic solution), followed by the addition of ultrapure water until a limpid monophasic solution was attained. This procedure was performed under constant stirring. The composition of the ternary systems was determined by weight quantification (± 10^–4^ g) of all components. All phase diagrams were correlated using Equation (1), proposed by Merchuk et al.^44^

(1) $$\left[\mathrm{IL}]=A\;\exp[(B{\left[\mathrm{Salt}\right]}^{0.5})-(C{\left[\mathrm{Salt}\right]}^{3})\right]$$

where [IL] and [Salt] are the IL and salt weight percentages (wt%), respectively, and *A*, *B*, and *C* are parameters obtained by regression of the experimental data.

For the determination of the tie‐line (TL), which are straight lines that describe the composition of the coexisting phases of a given mixture point, a ternary mixture composed of IL, Na_2_SO_4_, and H_2_O (35, 10, and 55 wt%, respectively) was gravimetrically prepared. After the complete separation of the phases, each phase was individually weighted. Then the TL was calculated by the resolution of the following four equation systems (Equations (2)–(5)).^44^ The compositions of the top and bottom phase are determined by the lever‐arm rule.

(2) $${\left[\mathrm{IL}\right]}_{T}=A\;\exp[(B\times {\left[\mathrm{Salt}\right]}_{T}^{0.5})-(C\times {\left[\mathrm{Salt}\right]}_{T}^{3})]$$

(3) $${\left[\mathrm{IL}\right]}_{B}=A\;\exp[(B\times {\left[\mathrm{Salt}\right]}_{B}^{0.5})-(C\times {\left[\mathrm{Salt}\right]}_{B}^{3})]$$

(4) $${\left[\mathrm{IL}\right]}_{T}=\frac{{\left[\mathrm{IL}\right]}_{M}}{\alpha }-\frac{1-\alpha }{\alpha }\times {\left[\mathrm{IL}\right]}_{B}$$

(5) $${\left[\mathrm{Salt}\right]}_{T}=\frac{{\left[\mathrm{Salt}\right]}_{M}}{\alpha }-\frac{1-\alpha }{\alpha }\times {\left[\mathrm{Salt}\right]}_{B}$$

where T, B, and M designate the top phase, the bottom phase, and the initial mixture, respectively. [IL] and [Salt] are IL and Na_2_SO_4_ weight fraction. The parameter α is the ratio between the top phase weight and the total mixture weight.

The solution of the referred system gives the concentration of IL and inorganic salt in the top and bottom phases.

The TLL denotes the distance, i.e., the difference in composition between the top phase and the bottom phase and was calculated according to Equation (6):

(6) $$\mathrm{TLL}=\sqrt{{\left({\left[\mathrm{Salt}\right]}_{T}-{\left[\mathrm{Salt}\right]}_{B}\right)}^{2}{}_{T}+{\left({\left[\mathrm{IL}\right]}_{T}-{\left[\mathrm{IL}\right]}_{B}\right)}^{2}}$$

*Organic Redox Molecules Partitioning*: The partition coefficient (*K*) represents the equilibrium distribution ratio of the target molecule between the top and the bottom phase and is defined by Equation (7):

(7) $$K=\frac{{\left[\mathrm{target}\;\mathrm{molecule}\right]}_{\mathrm{top}\;\mathrm{phase}}}{{\left[\mathrm{target}\;\mathrm{molecule}\right]}_{\mathrm{bottom}\;\mathrm{phase}}}$$

In order to determine the partition coefficients of each organic molecule, ternary mixtures were prepared in the biphasic region with the following composition Na_2_SO_4_ salt (10 wt%) + IL (35 wt%) + aqueous solution containing 20 × 10^−3^ m of the target molecule (55 wt%). Then, the systems were allowed to equilibrate for at least 12 h. After that, each phase was carefully separated and the redox molecules quantified by UV spectroscopy (using a BioTeck Synergy HT microplate reader at a wavelength of 240 nm for TEMPO, 314 nm for QUI, 329 nm for AQ2S, 258 nm for MV, and 290 nm for H_2_Q), using calibration curves previously established.

*Preparation of Redox‐Active ABS*: Redox‐active ABS is defined as a biphasic liquid–liquid system, in which two redox active molecules are selectively dissolved to form the catholyte and anolyte of a battery. Redox‐active ABS composed of Na_2_SO_4_, IL, and water (10, 35, and 55 wt%, respectively) were prepared adding two redox molecules with suitable partition coefficients at 20 × 10^−3^ m concentration each. Once the equilibrium between the two phases was established, their electrochemical properties as redox electrolytes were evaluated separately by means of electrochemical methods.

*Electrochemical Characterization*: The electrochemical behavior of each redox phase was analyzed by CV in a three electrode cell. A glassy carbon electrode and a platinum mesh were used as working electrode, and counter electrode, respectively. The reference electrode was a commercial Ag/AgCl (3 m NaCl) equipped with ceramic frit. All the measurements were performed at room temperature (circa 25 °C) and at 10 mV s^−1^ as scan rate in a Biologic VMP multichannel potentiostat.

The battery was assembled by mixing the same volume (4 mL) of each immiscible redox electrolyte in a cylindrical glass cell of 2.5 cm of diameter. No ion‐exchange membrane or physical separator was used to separate the two redox electrolytes. One carbon felt electrode (area 1.5 cm^2^) purchased from SGL CARBON GmbH (grade GFD 4.6 EA, 4.6 mm thickness) was introduced in each phase. The distance between the electrodes was 2 cm. One Ag/AgCl reference electrode was immersed in each immiscible redox phase as represented in Figure S4 in the Supporting Information. This membrane‐free battery was galvanostatic charged up to 20% of SOC and consecutively discharged at 0.16 mA cm^−2^. CE, VE and EE were calculated by Equations (8)–(10):

(8) $$\mathrm{CE}=\frac{Q_{d}}{Q_{c}}$$

(9) $$\mathrm{VE}=\frac{V_{d}}{V_{c}}$$

(10) EE=CE⋅VE

## Conflict of Interest

The authors declare no conflict of interest.

## Supporting information

Supplementary
