# Supplementary material for: Pioneering Use of Ionic Liquid‐Based Aqueous Biphasic Systems as Membrane‐Free Batteries
Source: Adv Sci (Weinh). 2018 Aug 8;5(10):1800576. doi: 10.1002/advs.201800576 (PMC6193149; doi:10.1002/advs.201800576)
Supplement: Supplementary file 1 — Supplementary [file ADVS-5-1800576-s001.pdf]

Copyright WILEY-VCH Verlag GmbH & Co. KGaA, 69469 Weinheim, Germany, 2018.

ADVANCED SCIENCE
Open Access

Supporting Information

for *Adv. Sci.,* DOI: 10.1002/advs.201800576

Pioneering Use of Ionic Liquid-Based Aqueous Biphasic Systems as Membrane-Free Batteries

Paula Navalpotro, Catarina M. S. S. Neves, Jesus Palma, Mara G. Freire, João A. P. Coutinho, and Rebeca Marcilla**

Supporting Information

Pioneering use of Ionic Liquid-based Aqueous Biphasic Systems as Membrane-Free Batteries

Paula Navalpotro, Catarina M. S. S. Neves, Jesus Palma, Mara G. Freire, João A. P. Coutinho Rebeca Marcilla**

Table S1. Experimental weight fraction data for the ABS composed of IL (1) + Na_2SO_4 (2) + H_2O (3) at (25 ± 1)°C and atmospheric pressure

**[P_{4444}]Br**		**[N_{4444}]Br**		**[P_{44414}]Cl**		**[P_{4444}]CF_3CO_2**	
**100 w_1**	**100 w_2**	**100 w_1**	**100 w_2**	**100 w_1**	**100 w_2**	**100 w_1**	**100 w_2**
76.0416	0.5827	70.5266	0.9226	77.7875	0.5163	64.5195	0.7558
57.1419	0.8702	53.0932	1.4811	67.9881	1.0010	21.3676	0.4425
53.1714	1.1917	45.6478	2.1867	54.6428	1.2530	19.1751	0.6022
49.5426	1.4947	42.2385	2.2860	52.2440	1.7239	16.9889	0.7135
46.2156	1.7927	39.4644	2.6387	48.4836	2.2617	16.1704	0.9283
44.0716	2.0683	37.9119	3.0684	45.8706	2.4731	15.4688	1.1012
41.8956	2.3203	35.6525	3.4500	43.9138	2.9243	14.6183	1.3163
40.0971	2.4473	34.8690	3.5500	41.0066	3.5717	13.8178	1.4557
38.7917	2.7243	33.8901	3.7847	38.9789	3.8181	13.3562	1.4972
37.1008	2.9805	32.9270	4.0631	36.9146	4.0072	12.6652	1.6090
36.0443	3.2123	32.1164	4.2620	35.8923	4.2282	12.1517	1.8152
34.7007	3.3751	31.4217	4.3865	34.8870	4.4466	11.3996	1.9558
33.8652	3.5922	29.7601	4.8531	34.2031	4.5973	11.0193	2.2139
33.0874	3.7434	28.7320	5.2710	33.2489	4.7587	10.6211	2.3835
32.2844	3.9201	27.3902	5.8728	32.0912	5.0947	10.3603	2.4843
31.6342	4.0714	25.9829	6.2356	31.2656	5.2223	10.0249	2.6056
30.9671	4.2169	25.2746	6.4822	30.7176	5.4409	9.8097	2.7934
30.2561	4.3454	23.8125	7.1264	30.2124	5.6099	9.4666	3.0156

29.5927	4.4754	23.1106	7.3774	29.5830	5.7229	9.0835	3.1802
28.6494	4.7670	22.2590	7.7877	28.5740	6.0634	8.6998	3.4926
28.0238	4.8944	21.2621	8.3267	27.3485	6.5699	8.4060	3.5908
27.4836	5.0192	19.4724	9.0055	26.6159	6.6383	8.0282	3.8784
26.6511	5.3037	18.0635	9.6974	25.7872	6.9143	7.6409	4.2080
26.1236	5.4095	17.1980	10.1396	25.1373	7.1038	7.3008	4.4243
25.3226	5.6687	16.0636	10.7451	24.3917	7.3473	7.0751	4.5592
24.4060	5.8638	15.0388	11.3004	23.7924	7.5417	6.8987	4.7435
23.7291	6.1011	14.2384	11.7025	22.8776	7.9455	6.7222	4.8622
22.6068	6.4275	13.8911	11.8853	22.2268	8.1529	6.5080	5.1017
21.6711	6.7074	13.2753	12.2148	21.8015	8.2184	6.3355	5.2528
20.7534	6.9675	12.5771	12.6086	21.2983	8.3737	6.2003	5.4231
19.9331	7.2042	12.2397	12.8124	20.7735	8.5680	6.0622	5.5250
19.4470	7.3843	11.5740	13.2055	20.0898	8.8750	5.9945	5.5968
18.7182	7.6077	11.1941	13.4506	19.7543	8.9293	5.8286	5.7641
18.3162	7.7461	10.5851	13.7876	19.1548	9.1614	5.6991	5.9206
17.9326	7.8797	10.2463	14.0419	18.7297	9.3122	5.4404	6.2140
17.6900	7.9180	9.7049	14.3726	18.3205	9.4655	5.2304	6.4346
17.3327	8.0473	9.1335	14.7616	17.7938	9.7145	5.1202	6.5765
17.0159	8.1659	8.7503	15.0218	17.4196	10.9491	5.0090	6.8628
16.8050	8.2003	8.3231	15.3271	17.1235	10.9714	4.8336	7.0384
16.4917	8.3116	8.1205	15.4725	16.7682	11.0565	4.6743	7.2279
16.1744	8.4290	7.5970	15.8709	16.3080	11.2645	4.4838	7.6148
15.8764	8.5303	6.8672	16.4982	15.7537	11.3777	4.1889	8.0460
15.5685	8.6485			15.3744	11.5608		
15.2964	8.7527			14.8516	11.7250		
15.0058	8.8602			14.1825	12.0069		
14.7480	8.9647			13.5656	12.2561		
14.4869	9.0610			13.0810	12.4397		
14.2331	9.1588			12.4947	12.7397		

13.9987	9.2517
13.6985	9.3927
13.4830	9.4623
13.2559	9.5495
13.0463	9.6325
12.7624	9.7633
12.2483	10.0065
11.9940	10.1278
11.7489	10.2494
11.5202	10.3553
11.2980	10.4518
11.0809	10.5518
10.8770	10.6553
10.6220	10.8012
10.4299	10.8964
10.2026	11.0219
10.0236	11.1105
9.8091	11.2313
9.5975	11.3514
9.3998	11.4619
9.2076	11.5689
9.0280	11.6688
8.6407	11.9048
8.4414	12.0323
8.0656	12.2760
7.6564	12.5538
7.2366	12.8498
6.7462	13.2405
6.2438	13.6538
5.5389	14.2850

12.0407	12.9407
11.2242	13.5107
10.4527	14.0216
9.9478	14.1936
9.3760	14.5200
7.9482	15.8233
5.8407	

Table S2. Parameters *A*, *B* and *C* obtained from Merchuk fitting [21] (equation (1) for each ABS tested.

IL	***A ± σ***	***B ± σ***	**10^5*(C± σ)***	***R^2***
$[C_4mim][CF_3SO_3]$	155.2 ± 8.3	-0.950 ± 0.033	4.97 ± 0.10	0.996
$[C_4mim][N(CN)_2]$	78.2 ± 1.7	-0.402 ± 0.015	31.56 ± 5.24	0.991
$[P_{44414}]Cl$	105.0 ± 2.9	-0.520 ± 0.014	13.10 ± 2.07	0.998
$[P_{4444}]Br$	95.2 ± 1.0	-0.538 ± 0.006	33.18 ± 1.21	0.985
$[N_{4444}]Br$	81.3 ± 1.7	-0.441 ± 0.010	15.00 ± 8.56	0.972
$[P_{4444}][CF_3CO_2]$	31.1 ± 0.9	-0.690 ± 0.022	8.55 ± 12.78	0.997

Table S3. Experimental data for TLs and TLLs of IL + Na_2SO_4 aqueous biphasic systems. Length of Tie line (TLL) and α were obtained from equation 2-6. pH values of all the studied IL-based ABS.

**IL**	***Weight fraction composition / wt%***						
	**$[IL]_T$**	**$[Salt]_T$**	**$[IL]_B$**	**$[Salt]_B$**	**TLL**	**α**	**pH**
$[C_4mim][CF_3SO_3]$	53.17	1.27	0.36	27.67	59.04	0.649	4.6
$[C_4mim][N(CN)_2]$	54.83	0.97	0.24	26.03	60.06	0.648	8.2
$[P_{44414}]Cl$	61.51	0.86	0.89	24.16	64.94	0.551	4.4
$[P_{4444}]Br$	64.74	0.51	0.18	22.35	68.15	0.543	3.1
$[N_{4444}]Br$	54.29	1.11	1.05	26.23	58.87	0.650	4.9
$[P_{4444}][CF_3CO_2]$	22.79	0.72	$6.01 \cdot 10^{-16}$	61.55	64.97	0.439	11.6

Table S4. Partition coefficients (*K*) calculated according to equation 7 as the ratio of concentrations of each compound in the two immiscible phases of all the studied acidic-neutral Na_2SO_4-based ABS and octanol-water system.

IL+ Na_2SO_4 + H_2O	TEMPO	H_2Q	AQ2S	QUI	MV
[C_4mim][CF_3SO_3]	$3.6\cdot10^{-3}$	0.21	0.02	0.14	2.89
[C_4mim][$N(CN)_2$]	3.56	13.53	98.86	11.49	0.72
[P_{44414}]Cl	6.55	3.39	13.15	10.31	0.02
[P_{4444}]Br	84.19	16.47	16.95	226.66	0.09
[N_{4444}]Br	55.86	4.05	12.43	120.00	0.13
[P_{4444}][CF_3CO_2]	3.96	2.08	25.89	185.22	$5.3\cdot10^{-3}$
**octanol -water (K_{ow})**	125.89	3.89	1.55	20.89	$1.99\cdot10^{-7}$

Table S5. Selectivity (*S*) calculated as the ratio of partition coefficient of the target molecule and MV ($K_{molecule}/K_{MV}$) in each ABS.

IL+ Na_2SO_4 + H_2O	H_2Q	AQ2S	QUI	TEMPO
[C_4mim][CF_3SO_3]	$7.41\cdot10^{-2}$	$0.60\cdot10^{-2}$	$4.67\cdot10^{-2}$	$0.12\cdot10^{-2}$
[C_4mim][$N(CN)_2$]	18.89	138.07	16.05	4.97
[P_{44414}]Cl	138.26	536.80	420.91	267.35
[P_{4444}]Br	178.44	183.57	2455.69	912.13
[N_{4444}]Br	30.45	93.46	902.25	420.00
[P_{4444}][CF_3CO_2]	391.70	4884.91	34947.16	747.55

Table S6. Potential redox reaction of majority specie in each phase of a system based on $[P_{44414}]Cl+Na_2SO_4$

**Combination**	**TOP Phase**					**BOTTOM Phase**					**OCV**
**Active Species (A.S)**	**A.S. Majority**	**E^0 (V*)**	**E_{pa} (V*)**	**E_{pc} (V*)**	**ΔE_p (V)**	**A. S. Majority**	**E^0 (V*)**	**E_{pa} (V*)**	**E_{pc} (V*)**	**ΔE_p (V)**	**(V)**
**MV-AQ2S**	AQ2S 1st step<br>2nd step	-0.57<br>-0.76	-0.53<br>-0.73	-0.61<br>-0.79	0.08<br>0.06	MV 1st step<br>2nd step	-0.64<br>-0.85	-0.62<br>-0.77	-0.66<br>-0.93	0.04<br>0.16	-
**MV-QUI**	QUI	-0.94	-0.72	-1.15	0.43	MV 1st step<br>2nd step	-0.68<br>-0.91	-0.65<br>-0.76<br>-0.84	-0.71<br>-0.99	0.05<br>0.15	-
**MV-H_2Q**	H_2Q	0.31	0.56	0.06	0.50	MV 1st step<br>2nd step	-0.64<br>-0.86	-0.55<br>-0.8	-0.73<br>-0.92	0.18<br>0.12	1.16
**MV-TEMPO**	TEMPO	0.66	0.7	0.63	0.07	MV 1st step<br>2nd step	-0.67<br>-0.93	-0.63<br>-0.9	-0.72<br>-0.97	0.09<br>0.07	1.33<br>1.6

*Potential vs Ag/AgCl reference electrode

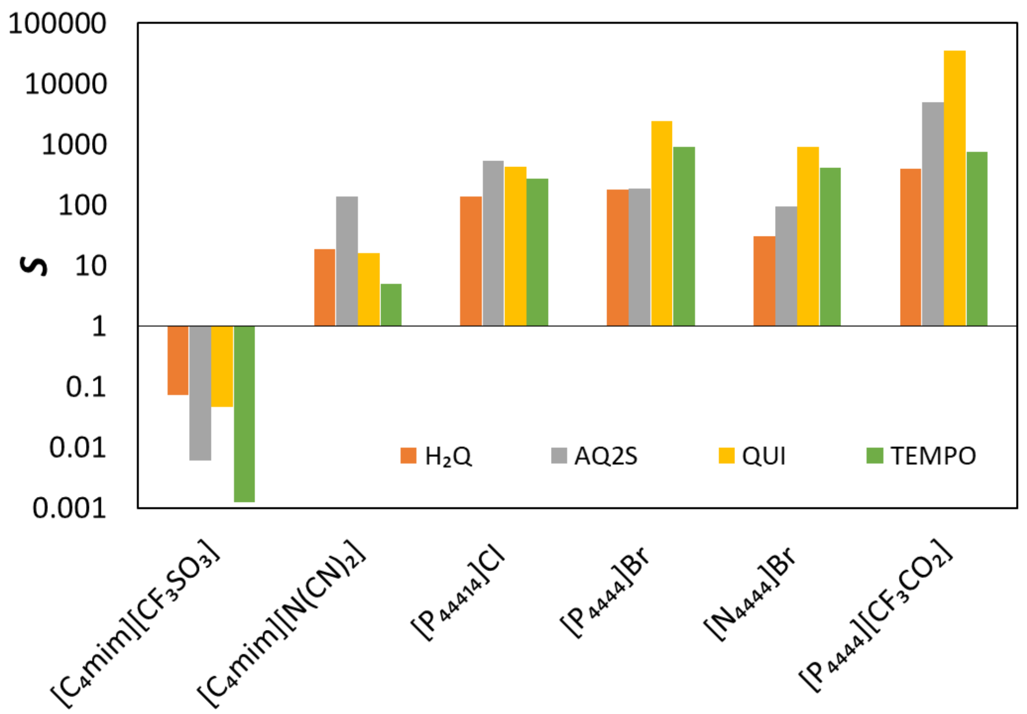

Figure S1. Selectivity of the target molecules with respect to MV

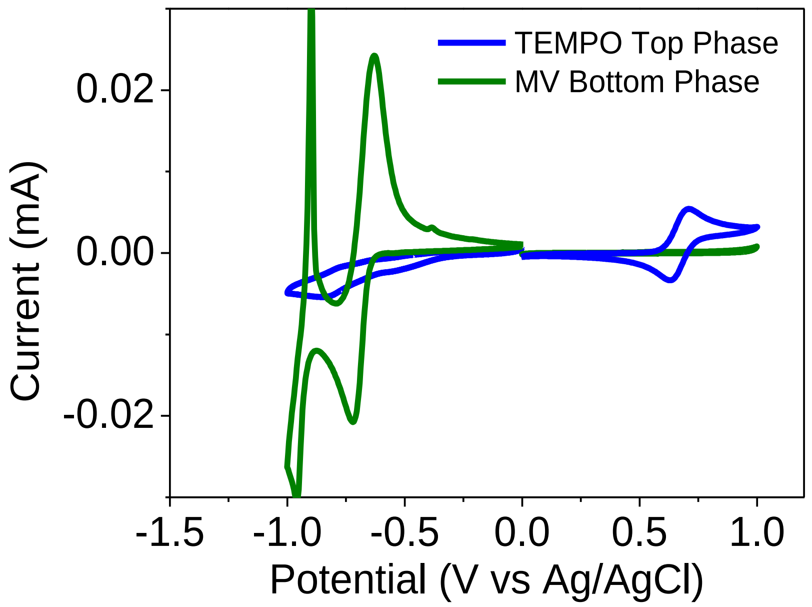

Figure S2. CV of each phase in the whole range of potential for the system based on $P_{44414}Cl$ with MV+TEMPO at 20 mM concentration.

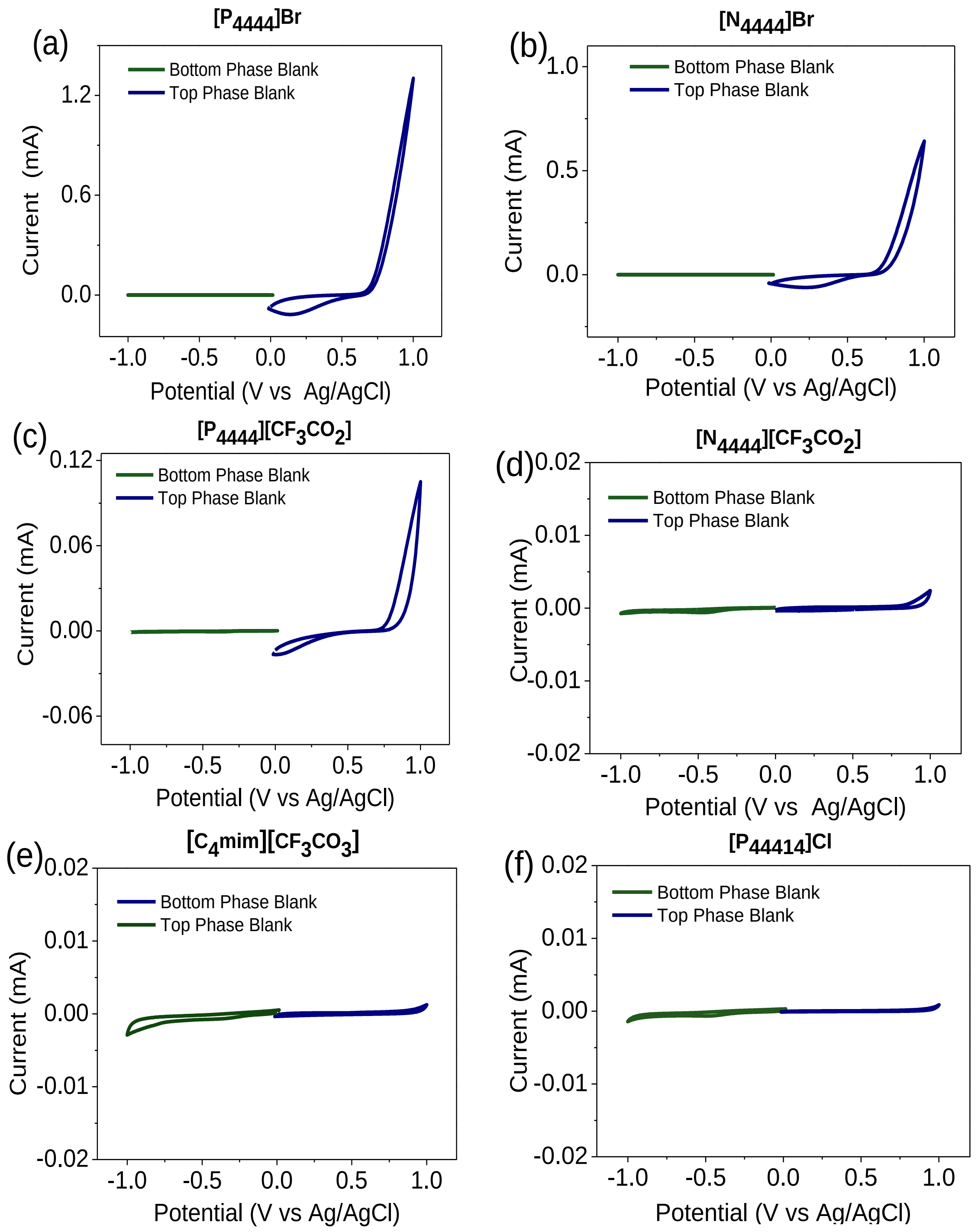

Figure S3. Cyclic voltammetry of separated phases of the IL-based ABS without actives species (blanks). (a) $[P_{4444}]Br$. (b) $[N_{4444}]Br$. (c) $[P_{4444}][CF_3CO_2]$. (d) $[N_{4444}][CF_3CO_2]$. (e) $[C_4mim][CF_3SO_3]$. (f) $[P_{44414}]Cl$. Scan rate $10 mVs^{-1}$.

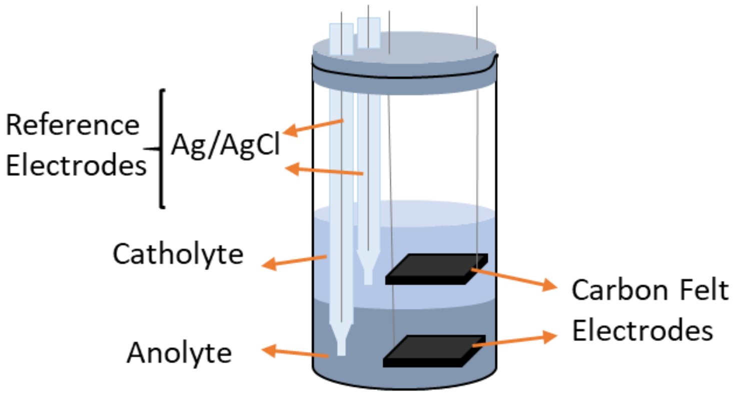

Figure S4. Schematic Illustration of the battery assembly.

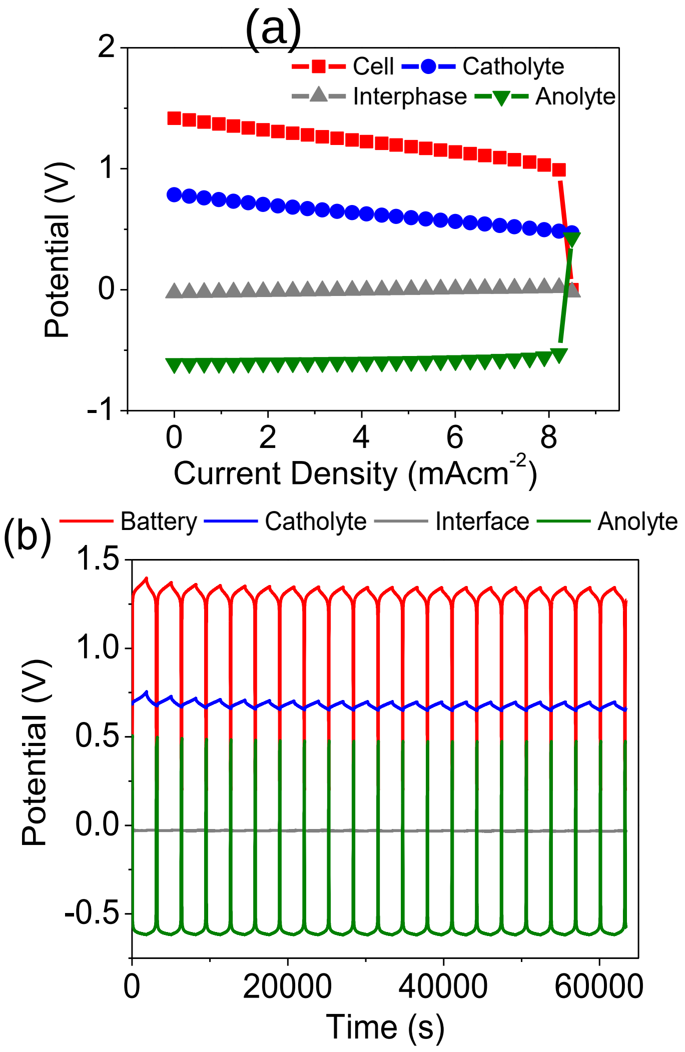

Figure S5. [P_{44414}]Cl-based Membrane-Free Battery. (a) Discharge Polarization curve. (b) Potential profiles of the battery, catholyte, anolyte and interface over cycling.
